# Reduced hippocampal activation during episodic encoding in middle-aged individuals at genetic risk of Alzheimer's Disease: a cross-sectional study

**DOI:** 10.1186/1741-7015-4-1

**Published:** 2006-01-13

**Authors:** Mehul A Trivedi, Taylor W Schmitz, Michele L Ries, Britta M Torgerson, Mark A Sager, Bruce P Hermann, Sanjay Asthana, Sterling C Johnson

**Affiliations:** 1 Geriatric Research Education and Clinical Center, William S. Middleton Veteran's Affairs Hospital, Madison, WI, USA; 2 Department of Medicine, University of Wisconsin Medical School, Madison, WI, USA; 3 Department of Neurology, University of Wisconsin Medical School, Madison, WI, USA

## Abstract

**Background:**

The presence of the apolipoprotein E (APOE) ε4 allele is a major risk factor for the development of Alzheimer's disease (AD), and has been associated with metabolic brain changes several years before the onset of typical AD symptoms. Functional MRI (fMRI) is a brain imaging technique that has been used to demonstrate hippocampal activation during measurement of episodic encoding, but the effect of the ε4 allele on hippocampal activation has not been firmly established.

**Methods:**

The present study examined the effects of APOE genotype on brain activation patterns in the medial temporal lobe (MTL) during an episodic encoding task using a well-characterized novel item versus familiar item contrast in cognitively normal, middle-aged (mean = 54 years) individuals who had at least one parent with AD.

**Results:**

We found that ε3/4 heterozygotes displayed reduced activation in the hippocampus and MTL compared to ε3/3 homozygotes. There were no significant differences between the groups in age, education or neuropsychological functioning, suggesting that the altered brain activation seen in ε3/4 heterozygotes was not associated with impaired cognitive function. We also found that participants' ability to encode information on a neuropsychological measure of learning was associated with greater activation in the anterior MTL in the ε3/3 homozygotes, but not in the ε3/4 heterozygotes.

**Conclusion:**

Together with previous studies reporting reduced glucose metabolism and AD-related neuropathology, this study provides convergent validity for the idea that the MTL exhibits functional decline associated with the APOE ε4 allele. Importantly, these changes were detected in the absence of meaningful neuropsychological differences between the groups. A focus of ongoing work in this laboratory is to determine if these findings are predictive of subsequent cognitive decline.

## Background

Family history of dementia and the apolipoprotein E (APOE) genotype are significant risk factors for the development of Alzheimer's disease (AD). The APOE gene, found on chromosome 19, has three allele variants (i.e., ε2, ε3 and ε4) and six possible genotypes, with the ε3/3 genotype being most prevalent in the general population (but see [1,2]). The presence of the ε4 allele significantly increases the risk and reduces the age of onset in people with the late-onset form of AD, the most common form of this disorder [1,3,4]. Previous studies have reported estimated cumulative lifetime risk percentages based on APOE genotype in individuals with a family-history of AD to be 30%, 46% and 61% for ε3/3 homozygotes, ε3/4 heterozygotes and ε4/4 homozygotes, respectively [5].

Impairment in the encoding and retrieval of episodic memories, presumably caused by neurodegeneration of the hippocampus and other medial temporal lobe (MTL) structures [6,7], is one of the earliest symptoms in AD [8]. Recent evidence suggests that healthy ε4 carriers (mean age = 56 years) with a family history of AD show a greater longitudinal, age-related episodic memory decline than non-carriers [9-11]. Furthermore, some structural MRI studies have found reduced hippocampal volume in older cognitively normal ε4 carriers [12-16] (but see [17-19]). Taken together, these results suggest that there may be an age-related phenotype for ε4 carriers involving cognitive decline and brain atrophy prior to the onset of AD. An important question is how early these changes occur and whether they can be detected *in vivo *using existing functional imaging methods.

The use of neuropsychological measures and brain imaging to examine cognitively normal individuals with AD risk factors such as family history or APOE genotype could potentially yield valuable information about preclinical alterations in neural function that precede the symptomatic stages of AD. Functional MRI (fMRI) is a non-invasive brain imaging technique that has been used successfully to demonstrate hippocampal and MTL activation during several tasks thought to reflect aspects of episodic memory [20-25]. The few studies that have examined brain activation differences between ε4 carriers and non-carriers have reported inconsistent findings on measures of episodic memory.

One episodic encoding paradigm that has been used to demonstrate robust hippocampal activation is novelty detection, in which individuals discriminate between events that were previously learned from events that are novel [26-31] (see [32,33] for reviews). Tulving and Kroll [34] have suggested that episodic encoding processes are more evident during the processing of novel information versus previously learned (or familiar) information. Therefore, novelty detection appears to be an important cognitive process involved in MTL-dependent episodic memory formation. Novel/familiar discrimination paradigms might therefore yield valuable information about functional changes in the MTL that precede overt changes in cognitive function in individuals at risk of AD.

The purpose of the present study was to use fMRI to test the hypothesis that cognitively normal ε3/4 heterozygotes with a family history of AD would display reduced activation in the hippocampus during a novel picture-encoding paradigm. We also used voxel-based morphometry (VBM) of T1 weighted images to determine whether any observed differences in fMRI activation between the groups were the result of differences in modulated GM volume. In addition, we compared hippocampal activation against a neuropsychological measure of episodic learning ability. Because prior research has shown that brain changes precede cognitive changes in this population, and because carriers of the ε4 allele may have altered hippocampal function, we hypothesized that there would be less systematic shared variance, disrupting the normal relationship between MTL activation and neuropsychological status in the ε3/4 heterozygotes as compared to ε3/3 homozygotes.

## Methods

### Subjects

Subjects who enrolled in this fMRI study were grouped by APOE genotype. The statistical analyses included 23 ε3/4 heterozygotes and 17 ε3/3 homozygotes. All participants in the study were recruited from the Wisconsin Registry for Alzheimer's Prevention (WRAP) [35], a longitudinal study designed to identify and evaluate factors that may delay or prevent the onset of AD. This cohort includes cognitively normal adult subjects (total N > 350 participants at the time of enrollment) between the ages of 40 and 65 with at least one biological parent who was diagnosed with AD by physicians affiliated with the Memory Clinics at the University of Wisconsin – Madison. The initial clinical diagnosis of AD in the parent was confirmed using published DSM-IV and NINDS-ADRDA criteria by a diagnostic consensus panel of experienced physicians and neuropsychologists. The adult children of these AD patients were then approached to participate in the WRAP study. The self-reported mean age at onset of memory problems in the parent diagnosed with AD was reported by the children to be 73 years old (range = 55 – 89 years).

All subjects in the WRAP underwent baseline medical laboratory tests, which included measurements of total non-fasting blood cholesterol, blood pressure, homocysteine, hemoglobin and hematocrit levels, and APOE genotyping. All participants also received detailed neuropsychological evaluations and baseline medical screening that included documentation of current medication usage. An important characteristic of this cohort is that 46% have at least one copy of the ε4 allele [35], compared to approximately 15% in the general population [2,36]. The battery of neuropsychological tests included the following: Wechsler Abbreviated Scale of Intelligence (WASI) verbal and performance IQ indexes [37], Wechsler Adult Intelligence Scale-III (WAIS-III) working memory index [38], the Controlled Oral Word Association Test (COWAT) [39], Rey Auditory Verbal Learning Test (RAVLT) [40], Boston Naming Test (BNT) [41], Trail Making Test A and B [40,42], and the Center for Epidemiological Studies Depression Inventory (CES-D) [43].

WRAP subjects were invited to participate in this fMRI study by direct mailings and newsletters. Subjects expressing interest in participating were contacted by phone and screened to determine if they met study eligibility requirements. The inclusion criteria consisted of the following: no current diagnosis of major psychiatric disease or other major medical conditions (e.g. diabetes, myocardial infarction or recent history of cancer), intact cognitive functions, and MRI scanner compatibility. Of the subjects included in this study, 55% had elevated (> 200 mg/dl) non-fasting total blood cholesterol (10 subjects in the ε3/3 group and 12 in the ε3/4 group) and 23% had elevated (> 140 mmHg) systolic blood pressure (5 subjects in the ε3/3 group and 4 in the ε3/4 group). There were no subjects in either groupwith high levels of homocysteine (> 14 μmol/l). Several participants were also taking a variety of medications, 4 subjects were using cholesterol-lowering medications (2 in the ε3/3 group and 2 in the ε3/4 group), 8 female subjects were current users of estrogen replacement therapy (5 in the ε3/4 group and 3 in the ε3/3 group), and 6 subjects were using selective serotonin reuptake inhibitors (5 in the ε3/4 group and 1 in the ε3/3 group).

Subjects included in the overall statistical analysis were required to have useable imaging data (movement in the x, y and z planes < 3 mm) and at least 90% accuracy on the fMRI memory task. We restricted our analyses to subjects with a homozygous ε3/3 or heterozygous ε3/4 APOE genotype to reduce the potential genetic variability in brain activation patterns, and because there was an insufficient number of individuals with other APOE genotypes (e.g. ε4/4 homozygotes or ε2/3 heterozygotes) to make meaningful inferencesregarding other APOE genotypes. The data from 6 additional subjects (3 ε3/3 homozygotes and 3 ε3/4 heterozygotes) were excluded from the overall statistical analyses: 1 subject for excessive motion during scanning, 1 subject for scanner error, 3 subjects who did not achieve 90% accuracy on the fMRI task, and 1 subject who had a previously undiagnosed tumor. The final number of subjects included in the statistical analysis was 40 (23 ε3/4 heterozygotes and 17 ε3/3 homozygotes). Athena Diagnostics (Worchester, MA) conducted APOE genotyping for all subjects using their patented procedures.

### fMRI task

Blood oxygen level dependent (BOLD) signal was detected using a variant of a well-known fMRI paradigm in which participants distinguished between novel and previously learned items. This paradigm is well-suited to examining potential differences in hippocampal activation based on APOE genotype because several previous PET and fMRI studies have demonstrated robust hippocampal activation for novel relative to familiar contrasts. The task consisted of serial presentations of line drawings obtained from the Snodgrass and Vanderwart [44] set that were matched for complexity and frequency. The previously learned pictures were presented in a training session 45 minutes prior to the task, and again during scanner setup 15 minutes prior to the task. The items were presented repeatedly in pseudorandom fashion for 15 exposures in each of the two training trials for a total of 30 exposures to each item. The participants were instructed to view the pictures and try to remember them.

During the fMRI test session, pictures were presented continuously at 3 s intervals for the entire experiment. For each picture, the participant decided whether the picture was previously learned or novel. Each picture was presented for 2800 ms with a 200 ms interstimulus interval. The novel pictures (condition 1) were intermixed with the presentation of previously learned pictures (condition 2) using a variable-length block (boxcar) paradigm. There were no periods of rest or fixation during the entire test session. Epoch length was variable, but appropriately balanced between conditions and ranged from single events to 5 consecutive items. The order of condition presentation and length were pseudorandom. Variable-length epochs rather than a fully event-related approach were used to reduce the condition predictability while maintaining some of the statistical power of the boxcar paradigm [45,46]. Two alternate forms of the task were presented (order counterbalanced), using the same previously learned items but different novel items. The duration of the entire task was 9 minutes and 24 seconds. Responses to the novel and previously learned pictures were made with a two-button response device held in the right hand. The index finger was used to identify previously learned pictures, whereas the middle finger was used to identify novel pictures. As in previous studies [see also [31]], we referred to encoding as the contrast of novel relative to familiar pictures.

### Imaging procedures

A GE 3.0 Tesla MRI scanner outfitted with an MR-compatible button-box and high-resolution goggles set at 800 × 600 (Resonance Technology; Northridge, CA) were used for fMRI imaging and stimulus presentation. Foam padding was placed around the head to reduce head motion. The software Presentation  was used to deliver visual stimuli and record responses in precise synchrony with slice acquisition and stimulus delivery. A T2*-weighted gradient-echo, echo-planar image (EPI) pulse sequence was obtained with higher order shimming during the functional trials for each subject.

The EPI parameters included: echo time flip angle = 90°; acquisition matrix = 64 × 64 voxels; field of view (FOV) = 240 mm; echo time (TE) = 30 ms; repetition time (TR) = 2000 ms. Thirty sagittal slices of the brain were acquired within the TR at each time point, with a voxel resolution of 3.75 × 3.75 × 4 mm and a 1-mm skip between slices. Over the entire test session, a total of 141 time points were collected. Three images acquired during the first 6 seconds of each scanning run were discarded. Following the functional scans, an axial T1-weighted inversion recovery prepared volume (124 slices; 1.2 mm thick; FOV 240 mm; matrix 256 × 256), and fast recovery fast spin echo T2 weighted anatomic images (TE 90 ms; 70 slices; 1.7 mm thick; 0.3 mm skip; FOV 240; matrix 256 × 256) were acquired. The images were later reviewed by a neuroradiologist for the presence of brain abnormalities that might exclude subjects from the statistical analyses. The T1 weighted images were also used for the VBM analysis.

### Functional image processing

The time series images were motion-corrected to reduce the effects of head movement during the scan session. 3 D field maps across the brain taken co-planar with the fMRI slices were used to correct distortions in the image files by measuring the phase of non-EPI gradient echo images at two echo times (7 and 10 ms). The continuous B field map was estimated using a 3 D phase-unwrapping algorithm based on Jenkinson et al. [47]. Image unwrapping was performed using a nonlinear pixel shifting and B splines interpolation algorithm. The images were then normalized into standard atlas space (using the T2* weighted template from SPM2), written out at a 2 × 2 × 2 voxel resolution, and then smoothed with an 8 mm full-width, half-maximum Gaussian kernel.

### Data analysis

Analyses of the time-series data were performed using the General Linear Model using the Statistical Parametric Mapping (SPM2) statistical software [48]. For each participant, the time-series statistical model included convolution with the canonical hemodynamic response function and a high frequency signal filtering (high pass filter = 128 seconds). Temporal autocorrelation was estimated using the first-order autoregressive (AR1) method on suprathreshold voxels. This method estimates the actual autocorrelation from the fMRI time series rather than imposing a generic temporal smoothing filter (see [49] for further discussion).

**Figure 1:**
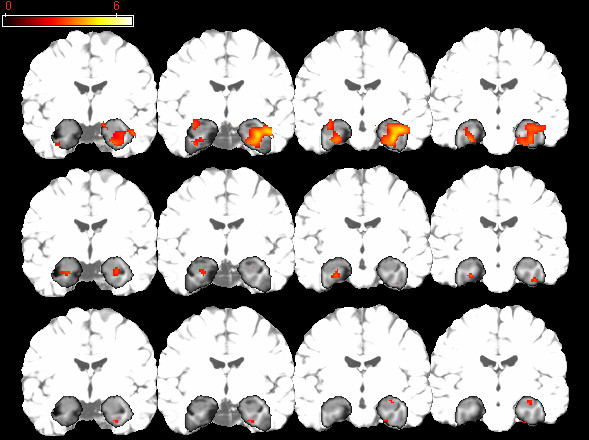
**Activation Maps for fMRI task**. Statistical parametric maps of the single-group analyses for ε3/3 homozygotes (panel A) and ε3/4 heterozygotes (panel B), and for regions where the ε3/3 homozygotes activated to a greater extent than the ε3/4 heterozygotes (panel C). The left side of each coronal section represents the left hemisphere. The dark-shaded area represents the MTL region to which the statistical analyses were confined. The lighter-shaded areasrepresent regions outside of the MTL mask.

The primary contrast used to evaluate group differences was novel versus familiar pictures. For completeness, we also examined group differences in activation for the familiar versus novel contrast. All single-subject analyses for both contrasts were computed for each participant and entered into a random effects group analysis. Each of the single-subject analyses was completed in the same semi-automated fashion regardless of APOE genotype. The random-effects group analysis and the correlational analyses were constrained to the right and left MTL, with an anatomical mask described previously by Johnson et al. [50]. The mask extended from the anterior aspect of the amygdala to the posterior aspect of the tail of the hippocampus, and also included the fusiform and parahippocampal gyri (See Figure 1). All possible voxels within each hemisphere of the MTL mask were considered potential dependent variables. The mask was used to impose more stringent hypothesis-driven restrictions on the number of simultaneous comparisons. Uncorrected p-values were used for this restricted region of interest (ROI) approach. In a second step, we conducted whole brain between-group analyses for both contrasts (p < 0.05; False Discovery Rate corrected for multiple comparisons) (see [51]).

### Voxel-based Morphometry processing steps

The detailed processing methodology for VBM has previously been reported in detail. Briefly, we used the optimized VBM approach described by Good et al. [52] (see also [53,54]) using SPM2. This procedure involves segmentation of the images into gray matter (GM), white matter (WM) and cerebrospinal fluid (CSF), and normalization of the GM images to the GM template in standard space. The GM images were then modulated using the Jacobian values derived from spatial normalization and smoothed with a 12 mm isotropic Gaussian kernel. The smoothed GM images were then entered into a random-effects two-sample t-test in SPM2 to examine GM volume differences between the groups.

## Results

### Neuropsychological functioning and fMRI task performance

The mean scores and standard deviations for the demographic and laboratory test variables and neuropsychological test performance are presented in Table 1. Between-group differences were examined using two-sample t-tests with a Bonferroni-corrected alpha level set at p < 0.002 (i.e., p < 0.05 divided by 22 comparisons). There were no significant differences between the groups in respect of age, education, laboratory test results or fMRI task performance. Both groups achieved at least 98% accuracy on the fMRI memory task and there were no differences in reaction times. Furthermore, there were no significant differences between the groups in terms of neuropsychological functioning or subjective memory complaints. The two-sample t-tests for each neuropsychological measure revealed no significant differences between the two groups for any of the neuropsychological test measures at the corrected threshold of p < 0.002. However, ε3/4 heterozygotes took longer to complete Trail Making Test B than ε3/3 homozygotes, but this finding was only significant using an uncorrected threshold of *p *< 0.05, whereas no significant differences between the groups were found for any of the other test measures. The performances of both groups on Trail Making Test B were within the normal range based on age-matched normative data from Spreen and Strauss (1998), as was performance on the rest of the neuropsychological test battery.

A follow-up analysis that included all WRAP subjects that were ε3/3 homozygotes (n = 154) and ε3/4 heterozygotes (n = 142), including those who did not participate in the fMRI study, revealed no significant difference on Trails Making Test B or any of the other neuropsychological measures. Therefore, the significantly slower time to complete Trails B in the ε3/4 heterozygotes who participated in the fMRI study does not appear to generalize to the WRAP cohort overall, and does not appear to be a general effect of the APOE ε4 allele.

### Imaging results

Figure 1 depicts MTL activation for the novel versus familiar contrast in the ε3/3 homozygotes and ε3/4 heterozygotes separately. The ε3/3 homozygotes show a greater level of activation for the novel versus familiar contrast in the MTL including the hippocampus compared to the ε3/4 heterozygotes.

**Figure 2:**
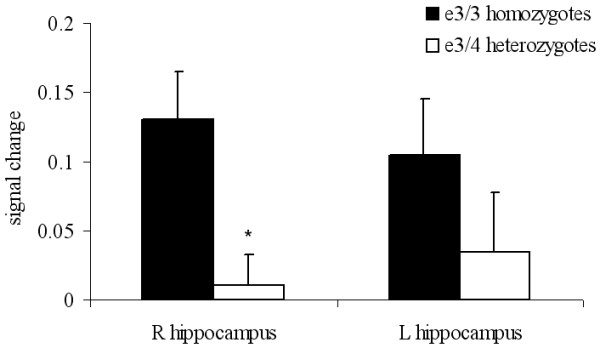
**Graph of signal change in the MTL**. Plot of the signal change (adjusted fitted responses) for ε3/3 homozygotes and ε3/4 heterozygotes averaged over a 2 mm radius spherical ROI at the local maxima in the right hippocampus (*x, y, z*: 30, -14, -16), and at the same location in the left hippocampus (*x, y, z*: -30, -14, -16). Data are presented as mean signalchange for each group. Error bars represent the standard error of the mean. * denotes significant difference between the two groups.

The single group analyses provide a qualitative description of differences in MTL activation between ε3/4 heterozygotes and ε3/3 homozygotes. To determine whether there were significant differences between the groups, a random-effects 2-group t-test (with age as a covariate) for the novel versus familiar contrast was performed in the MTL ROI. There was a significant main effect of APOE genotype (*x, y, z*: 30, -14, -16; *t *= 3.01, *p *= 0.002, uncorrected) with ε3/4 heterozygotes showing less activation for the novel versus familiar pictures contrast in the MTL than the ε3/3 homozygotes (see Figure 1). A plot of the mean signal change averaged over a 2 mm radius sphere in the hippocampus depicts the group difference (*x, y, z*: 30, -14, -16; see Figure 2). For completeness, we extracted mean signal change at the same location in the left hippocampus, and the ε3/3 homozygotes displayed numerically but not significantly (p > 0.25) greater signal change in the left hippocampus as well (see Figure 2). Table 2 provides statistics, locations and cluster size for all significantly activated regions for the genotype effect. There were no significant differences in MTL activation for the contrast of familiar relative to novel pictures.

The whole brain analyses for the novel vs familiar and familiar vs novel contrasts (p < 0.05; FDR corrected for multiple comparisons) revealed no significant differences between the groups for either contrast. In order to examine potential compensatory activity in the cortical regions of ε3/4 heterozygotes, we conducted a follow-up analysis (p < 0.01, uncorrected) using a two-sample t-test in which the analysis was constrained to include only those regions that were active for each contrast (i.e. novel versus familiar or familiar versus novel) collapsed across all 40 subjects (i.e. a one-sample t-test). In addition to reduced MTL activation, the ε3/4 heterozygotes also displayed reduced activation compared to the ε3/3 homozygotes for the novel versus familiar contrast in the right ventral temporal cortex and left parietal cortex. Importantly, the ε3/4 heterozygotes did not display greater activation compared to the ε3/3 homozygotes in any brain region even at the uncorrected threshold of p < 0.01. There were no differences between the groups for the familiar versus novel contrast.

To determine whether the significantly greater MTL activation displayed by the ε3/3 homozygotes relative to the ε3/4 heterozygotes was attributable todifferential MTL atrophy, we used VBM analysis [53] of the T1 weighted scans restricted to the MTL anatomical mask in these same participants. This analysis revealed no significant differences in modulated GM volume between the ε3/3 homozygotes and ε3/4 heterozygotes even at a threshold of p < 0.05, uncorrected. The absence of differential MTL atrophy indicates that the observed differences in fMRI activation were not attributable to GM volume differences in this group of subjects.

### Relationship between RAVLT and MTL activation

**Figure 3:**
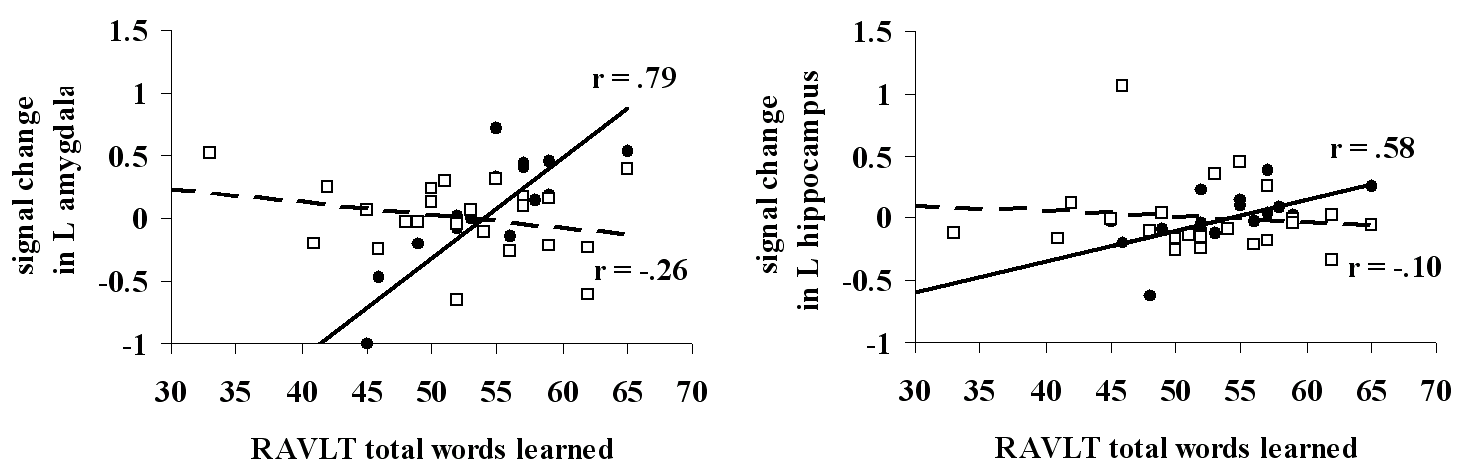
**RAVLT versus Signal Change**. Scatter plot of the association between total words learned on the RAVLT and signal change within the left amygdala (left panel) and left anterior hippocampus (right panel) for ε3/3 homozygotes (black circles, solid lines) and ε3/4 heterozygotes (white squares, hashed lines).

We also examined whether the total number of words learned on the RAVLT was associated with fMRI activation in the MTL for the novel relative to previously learned pictures contrast in ε3/4 heterozygotes and ε3/3 homozygotes. Since RAVLT variables are highly correlated, we chose to use total words recalled on the RAVLT because this measure has previously been shown to be sensitive to the preclinical detection of early AD [55]. These analyses revealed that total words learned on the RAVLT was positively associated (see Figure 3) with increased activation in the left anterior hippocampus (*x, y, z*: -28, -6, -26; *r *= 0.58; p < 0.01), and the left amygdala (*x, y, z*: -38, 4, -26; *r *= 0.78; *p *< 0.0001) in the ε3/3 homozygotes, but was not significantly associated with activation in the MTL in ε3/4 heterozygotes (r values = -0.26 and -0.10 for left amygdala and hippocampus, respectively). Furthermore, the magnitude of the correlations was significantly different between the two groups for both the left amygdala (p < 0.0001) and hippocampus (p = 0.01). In contrast, RAVLT total word recall was associated with activation in the left (*x, y, z*: -12, -38, 12; *r *= 0.65, *p *< 0.0001) and right (*x, y, z*: 4, -40, 4; *r *= 0.65, *p *< 0.0001) isthmus of the cingulate cortex in ε3/4 heterozygotes.

For completeness, we also determined the association between RAVLT total word recall and signal at the same coordinates in the right amygdala and hippocampus. These analyses failed to reveal a significant correlation between RAVLT total word recall and signal change in the right amygdala or hippocampus of the ε3/3 homozygotes (*r *values = 0.03 and 0.11 for the right amygdala and hippocampus, respectively) or the ε3/4 heterozygotes (*r *values = 0.19 and 0.11, for the right amygdala and hippocampus, respectively).

## Discussion

The present study examined the effects of APOE genotype on brain activation patterns in the MTL during an episodic encoding task in cognitively normal individuals with a family history of AD who were on average 15–20 years younger than the age at which AD symptoms typically develop. We found that ε3/4 heterozygotes displayed reduced fMRI activation compared to ε3/3 homozygotes in the right hippocampus and entorhinal cortex for the contrast of novel relative to familiar pictures. Importantly, there were no fMRI activation differences between the groups for the reverse contrast (i.e. familiar relative to novel), suggesting that the reduced activation found in the right hippocampus and MTL of ε3/4 heterozygotes was not caused by greater fMRI activation to the previously learned items in the ε3/4 heterozygotes, at least when measured relative to novel items. In subsequent whole-brain analyses, no brain regions were found to display greater activity in the ε3/4 heterozygotes than in the ε3/3 homozygotes for either contrast.

There were also no significant differences between the groups in age, education or memory function, and neuropsychological performance was within the normal range for both groups. This suggests that the reduced MTL activation to novel items (relative to familiar items) in ε3/4 heterozygotes was not caused by impaired cognitive function, and that the observed neurobiological changes in MTL function precede the onset of measurable decline in cognitive function.

We also found no evidence for differences in regional GM volume as measured by VBM, suggesting that the observed activation differences were not caused by reduced MTL GM volume in our cohort of middle-aged subjects. Previous volumetric studies have reported inconsistent findings regarding the effects of the ε4 allele on regional brain volume. Reiman et al. [56] reported nonsignificant trends towards smaller left and right hippocampal volumes in ε4/4 homozygotes (mean age = 58 years), and smaller hippocampal volumes were associated with reduced long-term memory ability. Den Heijer et al. [13] found that elderly ε4 carriers (mean age = 72 years) displayed significantly greater hippocampal and amygdalar atrophy and poorer memory ability relative to ε3/3 homozygotes. Moffat et al. [15] found that older ε4 carriers (mean age = 69 years) displayed a significantly greater rate of hippocampal volume loss over a 3-year follow-up period, although ε4 carriers were also found to display mild decline in memory ability over the same time frame. In a recent large-scale (N = 750) VBM study, Lemaitre et al. [14] found significantly reduced MTL (including hippocampus) volume in elderly (age range 63–75 years) ε4/4 homozygotes compared to both ε3/4 heterozygotes and non-carriers, whereas no significant differences were found between the ε3/4 heterozygotes and the non-carriers. Furthermore, these authors also found that the relative risk of cognitive impairment over a 4-year follow-up period was substantially greater in ε4/4 homozygotes relative to both ε3/4 heterozygotes and non-carriers. Other studies have also failed to demonstrate reduced regional brain volume in ε4 carriers [17-19]. The results of the present study are consistent with these findings.

Since the risk of memory impairment and AD is significantly greater in elderly ε4 carriers relative to non-carriers and younger ε4 carriers [57], cognitive status and age might interact with regional changes in brain volume. Therefore, it is quite possible that the MTL volume reductions found by previous studies in elderly populations were caused by the inclusion of APOE ε4 carriers who were more likely to be in the early stages of AD relative to non-carriers. This interpretation is supported by the findings of previous studies that reduced MTL volume in ε4 carriers was associated with poorer memory performance and increased risk for AD. In any case, the results of the present study failed to demonstrate significant differences in regional GM volume in middle-aged APOE ε3/4 heterozygotes, further supporting the notion that reduced fMRI activation in ε3/4 heterozygotes preceded overt changes in hippocampal volume. More studies are needed to determine the conditions under which the APOE ε4 allele results in reduced MTL volume.

In the present study, we also found that greater encoding ability on a neuropsychological measure of learning (i.e. RAVLT) was positively associated with fMRI activation in the left anterior MTL in the ε3/3 homozygotes but not in the ε3/4 heterozygotes. This signifies that among the noncarriers (in whom the MTL is presumably more intact) the strength of the MTL response was closely matched with better learning ability, whereas among the ε3/4 heterozygotes this relationship was disrupted. The amygdala and anterior hippocampus relationships found in ε3/3 homozygotes are consistent with a facilitative role for the amygdala in processing novel episodic information [58]. There was no significant difference in the magnitude of the correlations in the amygdala and hippocampus of the ε3/3 homozygotes, so conclusions regarding any differential contribution of these regions to encoding ability are limited. Future studies examining amygdala-hippocampal connectivity during encoding, as well as psychological factors (e.g. context, affective valence) that may modulate neural activity in these regions would be required to determine the role of the amygdala in encoding of novel visual information in people at risk of AD. Nevertheless, this finding does suggest that the left anterior MTL was recruited to a greater extent in ε3/3 homozygotes, whereas ε3/4 heterozygotes with better encodingability may have used encoding strategies that did not actively involve the anterior MTL. The fact that the correlation was found in the left hemisphere in ε3/3 homozygotes is consistent with previous studies that have reported primarily left MTL activation for encoding of verbal material (see [59] for review but see also [60]), suggesting that subjects with greater activation may have used a verbal encoding strategy. In contrast, previous studies in cognitively healthy young individuals (mean age = 30) have reported correlations between memory ability and right hippocampal signal change [60]. The exact reason for the divergent findings is not known. More studies examining correlations between memory ability and fMRI signal change in the left and right hippocampus during episodic encoding are needed to clarify these findings.

To date, six studies have examined brain activation differences between ε3/3 homozygotes and ε4 carriers using fMRI, but only two of these used an activation task that had an episodic encoding component. In a series of studies, Smith et al. [61-63] compared fMRI activation between a group of cognitively healthy subjects with a high risk of AD (i.e. ε4 carriers with a family history of AD) versus a low risk group (ε3/3 homozygotes with no family history of AD) during letter fluency and object naming tasks relative to a low-level, resting baseline condition (responding to a grayscale square that randomly changed in intensity). These authors found that ε4 carriers displayed increased activation in the left parietal region during a letter fluency task, and reduced activation in the inferotemporal cortex during the object-naming task. Longitudinal follow-up of a smaller subset (N = 25) of these participants four years later with the same object naming task revealed a greater longitudinal decline in fMRI activation in the inferotemporal cortex of the ε4 carriers compared to ε3/3 homozygotes. Since object naming and letter fluency are generally affected after episodic memory symptoms appear in AD [64], it may be that an episodic memory task would be more helpful in assessing the primary area of early pathology in AD – the MTL [6].

Bookheimer et al. [65] compared brain activation differences between cognitively normal ε4 carriers and ε3/3 homozygotes (age range: 47–82 years) using a paired-associate task as a probe of episodic memory. In their task, subjects were to encode seven unrelated word pairs over six learning trials, in which each learning trial was followed by 30-second periods of rest. The encoding phase was followed by six recall trials in which subjects heard the first word of each pair and were asked to recall the second word silently. The major contrast used to examine group differences was encoding + recall relative to the resting baseline. Whole-brain analysis revealed greater activation in the left prefrontal cortex, bilateral orbitofrontal, superior temporal, and inferior and superior parietal regions in ε4 carriers relative to the ε3/3 homozygotes for the contrast of encoding + recall relative to rest. In follow-up ROI analyses, these authors reported that ε4 carriers displayed greater signal change in the left MTL as well. In a follow-up study, Burrgren et al. [66] reported no differences between ε4 carriers and ε3/3 homozygotes during performance of a modified digit-span (forwards) working memory task relative to a baseline condition that was a single digit. They hypothesized that their results reflected a compensatory response in which ε4 carriers require additional cognitive effort to achieve comparable performance during episodic memory encoding tasks.

More recently, Bondi et al. [67] found that cognitively-healthy, older (mean age = 76) ε4 carriers displayed greater activation in the fusiform gyrus, parietal cortex and frontal gyrus compared to ε3/3 homozygotes using a paradigm in which subjects had to discriminate novel pictures from a single repeating picture. Follow-up ROI analysis revealed that ε3/3 homozygotes displayed greater activation in the left MTL compared to ε4 carriers, consistent with the findings of the present study. In contrast, the opposite pattern of results was found in the right MTL (i.e. ε4 carriers displayed greater activation in the right MTL). These authors also reported correlations between memory ability on a word-list learning task and right and left hippocampal activation during picture encoding (i.e. a positive correlation in ε3/3 homozygotes and a negative or zero correlation in carriers of the ε4 allele) that were similar to the correlations found in the present study. They suggested that their results were consistent with the compensatory response hypothesis described by Bookheimer et al. [65].

The exact reason for these contradictory findings is not knownp; however, there are several demographic and methodological differences between our study and the two previous fMRI studies that employed an episodic memory task. First, our subjects were on average 10 to 23 years younger than the participants in Bookheimer et al. [65] and Bondi et al [67], complicating comparisons between studies. Cabeza et al. [68] found that older individuals (mean age = 70 years) displayed reduced hippocampal activation during episodic retrieval, but greater activity in the prefrontal and parahippocampal cortices compared to young individuals (mean age = 23 years) [see also [69]]. Second, 60% of the subjects in Bookheimer et al. [65] had a family-history of AD, whereas 100% of our subjects had at least one biological parent with AD (Bondi et al. [67] did not report the percentage of subjects in his study with and without a family history of AD). Therefore, differences in these demographic variables might be responsible for the discrepant findings. Longitudinal studies similar to Smith et al. [63] that use episodic memory tasks and employ a 2 × 2 × 2 factorial design with family history of AD and APOE genotype as grouping variables are needed to help clarify these findings.

Third, it is possible that the divergent findings may have resulted from differences in the episodic memory task employed. In the present study, we employed a relatively straightforward novel/familiar discrimination paradigm as the probe of episodic encoding. In contrast, Bookheimer et al. [65] used a paired associate learning/recall task that was presumably more difficult than the paradigm used to define encoding in the present study [see [70]]. The paradigm used in the present study also differed from the encoding task used by Bondi et al. [67]. We presented familiar pictures in two separate trials before the fMRI scan. In contrast, first exposure to the repeating picture was during the fMRI task in the paradigm employed by Bondi et al. [67] and also by others e.g. [30], therefore some reduction in hippocampal signal during the presentation of the repeating novel stimulus may have occurred (see [50]).

Fourth, it is also possible that the compensatory response in ε4 carriers is more evident in elderly ε4 carriers rather than middle-aged ε4 carriers. Presumably, the negative effects of the APOE ε4 allele on hippocampal structure and function accelerate with increasing age. It is therefore possible that reduced hippocampal volume in elderly ε4 carriers [12-16,19,56,71] (but see [18]) might lead to the recruitment of structures to compensate for accumulating neuropathology in the MTL. Smith et al. [62] suggested that increased activation in ε4 carriers might be caused by a disruption in upstream elements of a functional network, resulting in decreased input from regions that are affected early in AD such as the MTL.

Finally, an important methodological difference between our study and previous studies was the baseline condition used. In the present study, we contrasted novel relative to familiar pictures, whereas previous studies reporting increased activation in ε4 carriers used lower level baseline conditions. Since most fMRI designs employ a subtraction method (activation – baseline), the baseline task chosen has a major effect on brain activation patterns observed. Using rest as a baseline condition may augment unintended effects in the experimental task such as language or sensorimotor processes, and increase inter-subject variability in brain activation during the baseline condition since there is less control over the mental state of the subject (i.e. what is the subject thinking about during the resting condition?).

To address this issue, Stark and Squire [72] designed an elegant study to examine the effects of several different baseline conditions including resting fixation on MTL fMRI activation during presentation of novel and familiar pictures. These authors found that MTL activation to novel and familiar pictures was significantly greater when an active task was used as a baseline (in their case, an odd/even number comparison task) than when passive rest was used as the baseline. In fact, several MTL regions were found to display increased activation during rest relative to familiar or novel pictures. In contrast, when the active baseline task was used, both novel and familiar pictures were associated with significant bilateral activity throughout much of the MTL. These authors suggested that using rest as the baseline condition may reduce, eliminate or even reverse the sign of activity during a cognitive task. At the very least, the lack of control over the mental state of subjects during the resting state makes it not ideal to serve as a baseline condition for comparison to cognitive tasks.

More recent evidence suggests that the hippocampus is coactive during the resting state with several cortical structures including precuneus and posterior cingulate cortex [73], and this resting state activation is disrupted in individuals with MCI and AD [74,75]. These new data make the previous findings of increased task-related activation relative to low-level baseline conditions in ε4 carriers more difficult to interpret, and argue against the use of resting baseline conditions in fMRI studies targeting these brain regions, especially in individuals with AD or at risk for AD.

Several positron emission tomography (PET) imaging studies have also reported reductions in resting state cerebral metabolic rate of glucose (CMRgl) in the MTL and cortical regions of AD patients and cognitively normal ε4 carriers between the ages of 20 and 65 years [76-82], suggesting different baseline neural activity in individuals at risk for AD. Reduced cerebral glucose utilization has also been shown to inhibit the induction of synaptic plasticity in the hippocampus of rats [83] and impair learning and memory on hippocampus-dependent tasks [84]. Prior research has demonstrated that CMRgl is coupled to regional cerebral blood flow (rCBF) [85,86], and both CMRgl [87] and rCBF [88,89] are coupled to the neural response. The current finding of reduced hippocampal BOLD signal during episodic encoding in ε3/4 heterozygotes is consistent with these studies, supporting the idea that reduced glucose metabolism would lead to reduced neuronal activation in the MTL during encoding. More research is needed to determine the relationship between reduced resting levels of CMRgl in ε4 carriers and task-related changes in the fMRI BOLD signal.

Reduced hippocampal activation in ε3/4 heterozygotes is consistent with several previous fMRI studies of memory encoding in patients with AD and MCI. For example, AD patients display reduced hippocampal activation compared to elderly controls during memory encoding [90,91], whereas no differences were found in the motor cortex during a sensorimotor task [90]. Machulda et al. [90] also reported similar findings in MCI patients [see also [92]]. Johnson et al. [50] found that elderly normal subjects displayed a hippocampal adaptation response (i.e. reduction in signal intensity) to repeating unfamiliar faces that was not displayed by age-matched patients with MCI. Taken together, these prior results indicate that MTL activation is reduced in AD and MCI patients with objective memory impairment relative to elderly, cognitively normal controls.

In a recent study, Dickerson et al. [93] examined differences in the extent of brain activation (i.e. number of contiguous as well as non-contiguous voxels), but not the magnitude of activation, during episodic encoding in AD patients and elderly individuals with and without subjective memory complaints who did not meet the clinical criterion for MCI [94]. These authors manually traced bilateral ROIs of the hippocampus and entorhinal cortex from each participant's structural MRI. They found that individuals with memory complaints displayed increased extent of activation in each of the four ROIs relative to age-matched subjects with no memory complaints. AD patients were found to display significantly less extensive activation relative to both groups. Furthermore, APOE ε4 carriers were found to display a greater extent of activation relative to non-carriers when collapsed across the memory complaints factor.

Dickerson et al. [93] suggested that increased extent of MTL activation early in the course of prodromal AD was followed by a subsequent decrease in the extent of activation as the disease progresses. An important caveat to these findings is that the individuals with memory complaints had significantly greater education and total word recall on a word-list learning task relative to individuals without memory complaints. Therefore, increased extent of activation may have been related to the paradoxically better encoding ability in individuals with memory complaints. This interpretation is supported by the finding of the present study that total word recall on the RAVLT was associated with increased magnitude of activation during episodic encoding, at least in the ε3/3 homozygotes. Previous studies have also reported similar findings [60,95,96], suggesting that individuals with better episodic memory ability display greater fMRI activation in the MTL during episodic encoding tasks.

Reduced neural function in the MTL of asymptomatic ε4 carriers may also lead to a greater age-related decline in episodic memory over time. Several studies have reported that older ε4 carriers display significantly greater longitudinal age-related decline in episodic memory functions than non-carriers [9-11,97,98]. For example, Caselli et al. [97] reported that cognitively normal ε4 carriers (mean age = 60 years) displayed significantly greater longitudinal decline during a three-year follow-up period for total word recall on the RAVLT and delayed recall of complex figures. Our findings that hippocampal activation during a novel-picture encoding task is reduced in ε3/4 heterozygotes, and that memory encoding ability is positively associated with anterior MTL activation in ε3/3 homozygotes but not ε3/4 heterozygotes, are consistent with previous findings that MTL-dependent memory processes decline at a greater rate in individuals who are ε4 carriers and have a family history of AD. These results leave open the possibility that ε4 carriers recruit other brain regions and/or rely on other compensatory psychological processes (e.g. verbal rehearsal of visual information) during encoding to compensate for early pathological changes in MTL structure and function [65,67].

Studies in transgenic mice expressing a human form of the APOE ε4 allele also support the negative effects of the ε4 allele on hippocampal function. For example, the presence of the APOE ε4 allele increases the production of β-amyloid (the main constituent of the senile plaques in AD) in cultured hippocampal neurons [99], reduces synaptic plasticity in the hippocampus [100], promotes β-amyloid induced blockade of plasticity in the hippocampus [101] and impairs hippocampus-dependent spatial memory [102,103]. Our results are consistent with these findings as well.

This study has several limitations. We did not include a low-level baseline condition (for the reasons discussed above), limiting our ability to compare and contrast our results directly to previous fMRI studies of APOE genotype. Furthermore, the findings of fMRI activation differences associated with APOE genotype were restricted to ε3/3 homozygotes and ε3/4 heterozygotes with a parental history of AD. Therefore, our results only generalize to individuals with these APOE genotypes and a family history of AD. More studies are needed to determine the effects of other APOE genotypes (e.g. ε4/4, ε2/3) and family history of AD on brain activation during memory encoding. Third, we also did not include a post-scan recognition memory test to determine if the novel pictures were encoded into memory. While conclusions regarding APOE genotype differences in encoding related processes cannot be made directly from this study, they can be inferred on the basis that novel information is more likely to be encoded than familiar information (see also [31]).

Fourth, several of the subjects in this study had elevated total blood cholesterol and/or high blood pressure. Importantly, there were no group differences in the percentage of subjects with these conditions, and these percentages were also no greater than observed prevalence rates in the general US population. Fifth, several subjects in each group were current users of medications that influence brain function and may affect the hemodynamic BOLD response. More studies are needed to determine the effects of these drugs and/or medical conditions on the fMRI signal. Finally, the subjects in this study were predominantly Caucasian, highly educated and female. Additional studies are needed to determine whether our findings can be confirmed in subjects with other demographic characteristics. Despite these limitations, the results of the present study provide converging evidence for the idea that the MTL displays functional decline associated with the APOE ε4 allele in individuals with a family history of AD.

If compromised MTL function continues to be observed in healthy ε4 carriers, this group of subjects may represent a good study population for novel treatments designed to delay the onset of or to prevent AD. More studies are needed to clarify inconsistent findings and to determine the reliability, validity and clinical utility of hippocampal activation paradigms for the early detection of AD. Although the results of the present study indicate cross-sectional differences in MTL activation based on APOE genotype, future studies employing longitudinal designs will be required to determine whether or not differences in MTL activation in individuals at genetic risk for AD can be used to improve the detection of incipient AD.

## Conclusion

This study found that APOE ε3/4 heterozygotes with a family-history of AD displayed reduced activation compared to ε3/3 homozygotes in the MTL during a novel picture-encoding task. Importantly, these changes occurred in the absence of cognitive differences between the groups. We also found that greater encoding ability on a neuropsychological measure of learning (i.e. RAVLT) was positively associated with fMRI activation in the left anterior MTL in the ε3/3 homozygotes but not in the ε3/4 heterozygotes. Together with previous studies reporting reduced glucose metabolism and AD-related neuropathology, this study provides convergent validity for the idea that the MTL exhibits functional decline associated with the APOE ε4 allele.

## Competing interests

The author(s) declare that they have no competing interests.

## Authors' contributions

MAT and TWS carried out the statistical analysis, drafted the manuscript and assisted in the acquisition of data. MLR, BMT and SA assisted in data acquisition and drafting of the manuscript. MAS and BPH assisted in data acquisition, interpreting the results and drafting the manuscript. SCJ drafted the manuscript, conceived of the study concept and design, assisted in the statistical analysis and interpretation of the results. All authors read and approved the final manuscript.

## Pre-publication history

The pre-publication history for this paper can be accessed here:



## Figures and Tables

**Table 1:** Demographic, neuropsychological and fMRI task performance data

	ε3/ε3 (n = 17)	ε3/ε4 (n = 23)
Age (years)	52.1 (6.8)	54.0 (5.6)
Age Range (years)	42 – 65	43 – 65
Education (years)	15.9 (2.1)	16.5 (2.6)
Gender Ratio: Female/Male	11/6	13/10
Handedness (% righthanded)	94%	96%
Homocysteine (μmol/l)	8.2 (1.8)	7.9 (1.5)
		
Blood Cholesterol (mg/dl) ^1^	215.8 (32.6)	204.9 (31.4)
Hematocrit (mL/dl)	40.0 (2.1)	40.6 (2.6)
Hemoglobin (g/dl)	13.8 (0.8)	13.9 (0.9)
Systolic Blood Pressure (mmHg)	131.5 (14.1)	132.6 (17.8)
		
WASI Verbal IQ	112.8 (8.6)	113.6 (10.5)
WASI Performance IQ	114.0 (8.4)	111.9 (8.5)
WAIS-III Working Memory	109.5 (14.5)	104.4 (13.5)
COWAT adjusted raw score	42.8 (10.9)	47.1 (10.0)
Boston Naming Test	56.6 (2.7)	56.6 (1.8)
RAVLT total recall trials 1–5	54.0 (5.2)	52.1 (7.5)
RAVLT short delay recall	11.6 (2.6)	11.0 (2.1)
RAVLT long delay recall	11.7 (2.3)	10.7 (2.9)
RAVLT recognition	14.1 (1.2)	14.0 (1.4)
Trail Making Test A (s)	27.1 (6.0)	27.0 (8.1)
Trail Making Test B (s)	55.4 (19.7)	67.0 (22.3)*
CES-D	4.2 (4.4)	4.1 (4.2)
		
Snodgrass Accuracy (%)	99.1 (1.2)	98.6 (2.2)
Snodgrass RT (sec)	0.81 (0.2)	0.89 (0.1)
Snodgrass Misses	0.3 (0.8)	0.7 (1.6)

Data are presented as mean (SD). * denotes significant differences (p < 0.05) between the groups. WASI = Wechsler Abbreviated Scale of Intelligence, WAIS = Wechsler Adult Intelligence Scale, COWAT = Controlled Oral Word Fluency Test, RAVLT = Rey Auditory Verbal Learning Test, CES-D = Center for Epidemiological Studies – Depression Inventory. See text for further discussion of test measures. ^1 ^= total non-fasting Blood Cholesterol

**Table 2:** Montreal Neurological Institute (MNI) Coordinates for peak activation differences (p < 0.01; uncorrected) for the random-effects group analysis between ε3/4 heterozygotes and ε3/3 homozygotes for the functional MRI encoding contrast of novel versus familiar pictures. k = cluster size

Contrast	Region (k)	Montreal Neurological Institute (MNI) coordinates			Peak t value	P (uncorrected)
		x	y	z		

Novel vs. Familiar						
ε3/3 > ε3/4	Right anterior entorhinal cortex (60)	22	2	-34	3.56	0.001
	- Right parahippocampal gyrus	22	-8	-34	2.99	0.002
	Right hippocampus (93)	30	-14	-16	3.01	0.002
	- Right hippocampus	36	-24	-6	2.99	0.003
